# 利用生物信息学、免疫组化分析肿瘤来源免疫球蛋白在非小细胞肺癌中的表达及临床意义

**DOI:** 10.3779/j.issn.1009-3419.2019.06.03

**Published:** 2019-06-20

**Authors:** 国辉 王, 雄涛 杨, 广迎 朱

**Affiliations:** 100000 北京，北京大学中日友好临床医学院；中日友好医院放射肿瘤科；中日友好医院呼吸中心；国家呼吸疾病临床医学研究中心 Peking University China-Japan Friendship School of Clinical Medicine; Department of Radiation Oncology, Center of Respiratory Medicine, China-Japan Friendship Hospital; National Clinical Research Center for Respiratory Diseases, Beijing 100000, China

**Keywords:** 肺肿瘤, 肿瘤来源免疫球蛋白, 预后, 机制, Lung neoplasms, Cancer-IgG, Prognosis, Mechanism

## Abstract

**背景与目的:**

经典免疫学理论认为，免疫球蛋白G（immunoglobulin G, IgG）仅由B细胞合成。近年来研究发现恶性肿瘤细胞也可以合成IgG（cancer-IgG）。本研究分析了cancer-IgG在非小细胞肺癌（non-small cell lung cancer, NSCLC）中的表达及临床意义，并初步探究其机制。

**方法:**

应用数据库分析IgG1重链编码基因（immunoglobulin heavy constant gamma 1, IGHG1）、免疫组化分析cancer-IgG在NSCLC中的表达及与预后的关系；基因富集分析（gene set enrichment analysis, GSEA）方法探究与IGHG1调控相关的信号通路。

**结果:**

Cancer-IgG在NSCLC中的表达量显著高于正常组织，与预后呈负相关，并与患者的临床分期（*P*=0.042）、T分期（*P*=0.044）和转移（*P*=0.007）密切相关。GSEA分析显示，IGHG1与细胞黏附、细胞因子相互作用和趋化因子信号通路相关。

**结论:**

在NSCLC中，cancer-IgG高表达是预后不良的因素，可能与促进肿瘤的侵袭转移相关。

## Abstract (en)

**Background and objective:**

It was believed that immunoglobulin G (IgG) was synthesized only by B cells. However, in recent years, researchers have found that a variety of cancer cells can also synthesize IgG (cancer-IgG) which promote the development of tumors. This study analyzed the expression and clinical significance of cancer-IgG in non-small cell lung cancer (NSCLC), and initially explored its mechanism.

**Methods:**

The expression of IgG1 heavy chain gamma 1 (IGHG1) and cancer-IgG were detected by bioinformatics and immunohistochemistry in NSCLC; The gene set enrichment analysis (GSEA) method was used to explore the signaling pathways involved in IGHG1 regulation.

**Results:**

The expression level of cancer-IgG in NSCLC was significantly higher than that in normal tissues. The high expression group had a poor prognosis and was associated with clinical stage (*P*=0.042), T stage (*P*=0.044) and metastasis (*P*=0.007). GSEA analysis showed that IGHG1 was associated with cell adhesion, cytokine interaction and chemokine signaling pathway.

**Conclusion:**

High expression of cancer-IgG in NSCLC is a poor prognosis factor, which may be related to the promotion of tumor invasion and metastasis.

肺癌已经成为全球恶性肿瘤发病率和死亡率的首要原因。据预测，2018年有180万肺癌患者死亡，占恶性肿瘤死亡人数的近1/5（18.4%）^[1]^。其中，非小细胞肺癌（non-small cell lung cancer, NSCLC）占肺癌的83%，主要包括腺癌、鳞状细胞癌、大细胞癌。75%的NSCLC患者诊断时已处于中晚期，治疗方式主要有手术、放化疗、靶向治疗以及免疫治疗，但是其5年生存率仍然低于30%^[2]^。因此，需要进一步研究NSCLC发生发展的分子作用机制，以改进其现有的临床诊疗现状。

经典的免疫学理论认为，免疫球蛋白G（immunoglobulin G, IgG）是免疫系统在抗原刺激下，由B淋巴细胞或记忆B细胞增殖分化成的浆细胞产生、可以与相应抗原发生特异性结合的物质，是体液免疫的重要效应分子。然而我国学者邱晓彦教授在国际上首次发现非B细胞，特别是肿瘤细胞也可以合成和分泌IgG，称为肿瘤来源免疫球蛋白（cancer-IgG）^[3]^，并发现cancer-IgG在肿瘤的发生发展中发挥着重要的生物学作用^[4-9]^。这一区别于经典免疫学理论的观点，得到了越来越多国内外学者的关注。Lee等^[10]^利用卵巢癌细胞系OC-3-VGH的裂解产物去免疫小鼠，得到了一株单克隆抗体RP215，通过识别IgG重链恒定区的一个特殊糖基化位点，可以特异性地识别cancer-IgG。Liao等^[9]^发现，RP215识别的cancer-IgG促进了肿瘤细胞的增殖、侵袭和转移，并且可以作为一个潜在的肿瘤干细胞标志物^[4]^。Tang等的研究也表明，RP215识别的cancer-IgG是通过其特殊的糖基化表位激活黏着斑通路来促进肺鳞癌的发生发展。IgG由两条重链和两条轻链构成，每条重链和轻链都由可变区和恒定区构成。IgG1重链恒定区的编码基因（immunoglobulin heavy constant gamma 1, IGHG1）的表达量与cancer-IgG的表达量呈正相关^[11]^。已有研究^[6, 7, 12-15]^表明，IGHG1在膀胱癌、前列腺癌、肾透明细胞癌、胰腺癌、结直肠癌等处于高表达的状态，其与诱导肿瘤细胞凋亡、肿瘤免疫逃逸相关。

本研究利用生物信息学和免疫组化的方法，分析cancer-IgG在NSCLC中的表达情况与预后生存以及临床病例特征的关系。采用基因富集分析方法（gene set enrichment analysis, GSEA），分析IGHG1高表达样本所富集到的信号通路，初步探讨cancer-IgG参与肿瘤发生发展的机制。

## 1 材料与方法

### 1.1 生物信息学分析在基因表达汇编（gene expression omnibus, GEO）

数据库（http://www.ncbi.nlm.nih.gov/geo）下载NSCLC相关数据集GSE30219^[16]^、GSE33532和GSE37745^[17]^的原始数据，采用稳健多芯片平均标准化（RAM）方法对表达谱数据进行标准化处理（图 1）。以IGHG1表达值的中位数为分组依据，分为高表达组和低表达组。利用GSE33532数据集，比较NSCLC与正常组织中IGHG1表达差异；通过基因表达谱动态分析在线网站（Gene Expression Profiling Interactive Analysis, GEPIA）^[18]^分析TCGA和GTEx数据库中IGHG1在LUAD和LUSC中的表达情况；运用GSE30219数据集中的生存信息，依据IGHG1的表达量，绘制生存曲线。利用*Kaplan-Meier* Plotter数据库（http://kmplot.com/analysis）^[19]^中的肺癌数据集进行在线生存分析。数据集GSE37745中的组织样本依据IGHG1表达量的中位值分为高表达组和低表达组采用GSEA3.0版本进行基因富集分析（http://software.broadinsitute.org/gsea/index.jsp）^[20]^。从GSEA网站MsigDB数据库中获取c2.cp.kegg.v6.2基因集为参照基因，按照默认加权富集统计方法进行富集分析。每次分析重复1, 000次。

**1:**
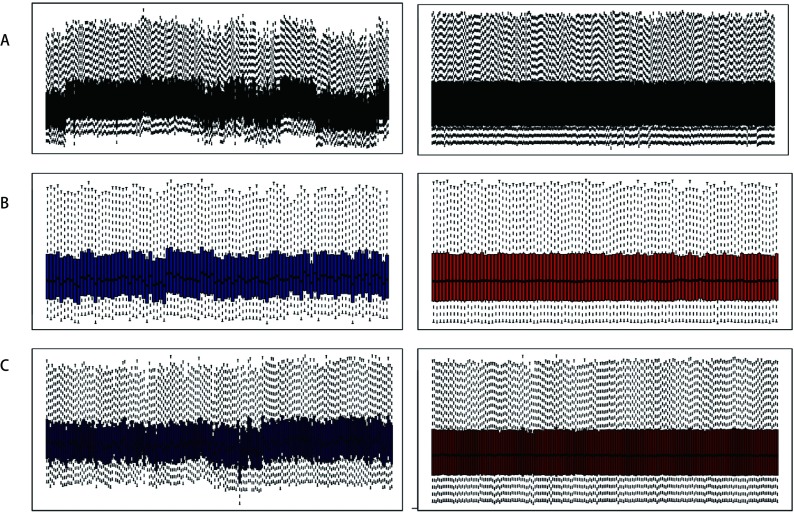
对GEO数据进行RAM标准化。A：GSE30219标准化前后对比图；B：GSE33532标准化前后对比图；C：GSE37745标准化前后对比图。 Standardization of gene expression. A:The standardization of GSE30219; B: The standardization of GSE33532; C: The standardization of GSE37745.

### 1.2 肺癌组织芯片及免疫组织化学染色肺癌组织芯片

购自上海芯超生物科技有限公司，所有患者经病理确诊为肺癌，收集性别、年龄、病理分级、T分期、淋巴结转移、远处转移和美国癌症联合会（American Joint Committee on Cancer, AJCC）第7版TNM分期等临床病理资料（表 1）。

**1:** RP215在NSCLC中的表达及临床特征分析 Association between RP215 expression and clinicopathological features of lung cancer patients

Characteristics	Number of cases (%)	RP215					*P*
		Low				High	
Age (yr)							0.729
≥60	26 (36.1)	8				18	
< 60	46 (63.9)	16				30	
Gender							1.000
Male	54 (75.0)	20			34		
Female	18 (25.0)	6			12		
Pathological type							0.632
Squamous	44 (61.1)	17			27		
Adenocarcinoma	28 (38.9)	9			19		
Clinical stage							0.042
Ⅰ	20 (27.8)	9		11			
Ⅱ	12 (16.7)	6		6			
Ⅲ	24 (33.3)	10		14			
Ⅳ	16 (22.2)	1		15			
T classification							0.044
T1	12 (16.7)	8	4				
T2	34 (47.2)	9	25				
T3	26 (36.1)	9	17				
N classification							0.594
N0	36 (50.0)	15				21	
N1	10 (13.9)	4				6	
N2	14 (19.4)	3				11	
N3	12 (16.7)	4				8	
Metastasis							0.007
No	56 (77.8)	24				32	
Yes	16 (22.2)	1				15	
NSCLC: non-small cell lung cancer.							

组织芯片置于二甲苯脱蜡20 min，更换新鲜二甲苯重复1次。将脱蜡后的芯片于100%乙醇中浸泡5 min 2次，95%乙醇、80%乙醇、蒸馏水各浸泡5 min。碱性抗原修复液（Tris-EDTA, pH=9）用高压锅加热至沸腾，将芯片放入，计时2 min，自然冷却至室温。3%H_2_O_2_室温下避光孵育10 min，用正常羊血清工作液室温封闭30 min。随后加入一抗RP215，4 ℃过夜，HRP标记的二抗室温30 min。DAB显色，苏木素复染。RP215单克隆抗体由北京大学医学部邱晓彦课题组馈赠，HRP标记的二抗购自于Cell Signaling Technology。

### 1.3 免疫组织化学染色判定每张切片

随机选取10个高倍（400×）视野，由两名病理科医生独立阅片。细胞质染色评分利用四个强度等级（0：阴性，1：弱阳性，2：中度阳性，3：强阳性）以及阳性细胞百分比等级（0: 0%, 1: 1%-5%, 2: 6%-25%, 3: 26%-50%, 4: 51%-100%）。最终的评分为强度等级和阳性细胞率（百分比）等级的乘积，0-3为低表达，4-9为高表达。

### 1.4 统计学分析

采用SPSS 20.0版统计学软件进行数据分析。不同组织中cancer-IgG表达水平的比较采用*χ*^2^检验及独立样本*t*检验，与临床病理特征分析采用*χ*^2^检验，生存分析采用*Kaplan-Meier*法并行*Log-rank*检验。*P* < 0.05定义为差异有统计学意义。在GSEA分析中，以*P* < 0.05及FDR < 0.25的基因集作为显著富集的基因集。

## 2 结果

### 2.1 IGHG1在NSCLC中的表达与临床预后之间的关系

在GSE33532数据集中，IGHG1在NSCLC中的表达水平为（6.635±0.125），正常肺组织的表达水平为（4.965±0.141），NSCLC中的表达量显著高于正常肺组织，差异具有统计学意义（*P* < 0.01）（图 2A）。分析TCGA和GTEx数据库中的LUAD和LUSC的数据，也得到了同样的结论：IGHG1在NSCLC中的表达量显著高于正常组织（*P* < 0.01）（图 2B）。分析GSE30219数据集中NSCLC患者的生存信息，IGHG1高表达组的总生存期较低表达组患者显著缩短（*P*=0.008, HR=1.632, 95%CI: 1.138-2.314）（图 2C）。再利用KM-plotter（http://kmplot.com/analysis）数据库在线分析IGHG1与NSCLC患者预后的关系，也得出了相似的结论，高表达IGHG1组的患者预后差于低表达组（*P*=3.4e-05, HR=1.4, 95%CI: 1.19-1.64）（图 2D）。

**2:**
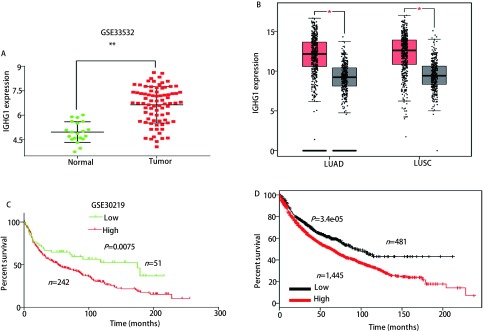
IGHG1在NSCLC中的表达情况及其与预后的关系。在GEO数据集GSE33532（A）和TCGA+GTEx数据库（B）中IGHG1在NSCLC中的表达显著高于正常肺组织（*P* < 0.01）。在GSE30219数据集（C）和*Kaplan-Meier* Plotter（D）中的NSCLC数据进行生存曲线分析，IGHG1高表达的患者预后显著差于低表达的患者。 IGHG1 expression in NSCLC and its correlation with prognosis. The expression of IGHG1 in NSCLC was significantly higher than that in normal lung tissues (*P* < 0.01) in GSE33532 (A) and TCGA+GTEx datasets (B); In the GSE30219 (C) and *Kaplan-Meier* Plotter (D) NSCLC with high expression of IGHG1 had significantly worse prognosis than with low expression.

### 2.2 Cancer-IgG在NSCLC中的表达与临床病例特征及预后的相关性

如图 3（A-D）所示，Cancer-IgG在胞膜、胞质和核膜上均有表达，阳性细胞呈巢状排列，在NSCLC中的阳性率为81.9%（59/72）。如表 1所示，依据cancer-IgG表达量的高低，进一步对该72例NSCLC患者的临床病例特征进行分析，结果显示：cancer-IgG与NSCLC患者的临床分期（*P*=0.042）、T分期（*P*=0.044）和转移（*P*=0.007）相关。生存分析显示，高表达cancer-IgG组中的总生存期显著低于低表达组（*P*=0.008, HR=1.746, 95%CI: 1.39-1.94）。

**3:**
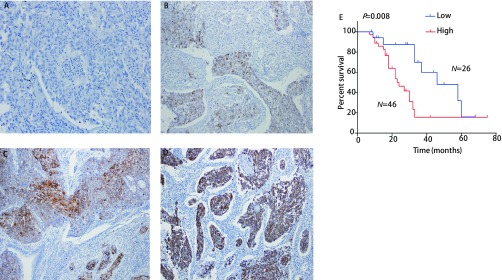
Cancer-IgG免疫组化示意图及与预后的相关性（×100）。Cancer-IgG在NSCLC中阴性表达（A）、弱阳性（B）、中度阳性（C）和强阳性（D）表达情况；E：高表达cancer-IgG的NSCLC患者生存期显著低于低表达的患者。 The immunohistochemistry staining results of cancer-IgG in NSCLC and *Kaplan-Meier* survival analysis. The expression of cancer-IgG in NSCLC with negative (A)、weak positive (B), moderate positive (C) and strong positive (D); E: The survival of patients with high expression of cancer-IgG in NSCLC was significantly lower than patients with low expression.

### 2.3

IGHG1的功能基因富集选用GSE37745数据集，运用GSEA分析方法分析IGHG1表达水平对调控基因集富集的影响。结果显示IGHG1高表达的肿瘤样本可以富集在细胞黏附（NES=1.654、NOM *P*=0.007、FDR *q*=0.085）、细胞因子-细胞因子相互作用（NES=1.690、NOM *P*=0.001、FDR *q*=0.075）和趋化因子信号通路（NES=1.564、NOM *P*=0.019、FDR *q*=0.125）（图 4）。

**4:**
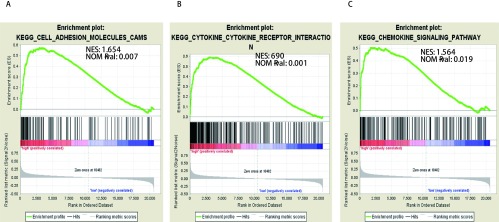
基于GSE37745样本的基因富集分析。GSEA结果显示IGHG1的高表达与细胞黏附分子（A）、细胞因子-细胞因子相互作用（B）和趋化因子通路（C）有关。 Enrichment plots from gene set enrichment analysis. GSEA results showed that high expression of IGHG1 was associated with cell adhesion molecule (A), cytokine-cytokine interaction (B) and chemokine pathway (C).

## 3 讨论

目前，经典的免疫学理论认为IgG仅由B淋巴细胞合成和分泌，是体液免疫的重要效应分子。IgG是由两个重链和两个轻链构成的Y形分子，轻链和重链之间以二硫键连接。每条重链和轻链都是由恒定区和可变区构成，可变区是由若干个基因片段所编码，包括可变段（V区）、多样段（D区）和连接段（J区）^[21]^。B淋巴细胞通过重排V、D和J基因片段，产生IgG的可变区。由于每段可变区基因都有不同的拷贝，各段之间还存在不同的组合方式，因此IgG的可变区可以发生数量巨大的变化，与抗原发生特异性结合，发挥抗体的功能^[22]^。我国学者邱晓彦教授在国际上率先发现肿瘤细胞也可以产生IgG，其结构与B淋巴细胞分泌的IgG最大的不同就是可变区呈现相对固定的重排模式。λ链的可变区主要呈现VH5-51/D3-9/JH4、VH3-30/D6-19/JH4两种重排模式，µ链的可变区主要呈现VH3-15/D3-10/JH4、VH6-1/D6-13/JH4和VH4-30-2/D3-22/JH4三种重排模式^[23]^。

Cancer-IgG在功能方面与B细胞来源的IgG也有很大的区别。Liao^[4]^发现在乳腺癌细胞中cancer-IgG与肿瘤细胞的侵袭和转移密切相关，高表达cancer-IgG的细胞具有肿瘤干细胞的特征。Liu^[13]^发现IgG1重链编码基因*IGHG1*在前列腺癌组织中的表达显著高于正常前列腺组织，并且与前列腺癌的组织学分级相关。顾江^[11]^等的研究证明了IGHG1与cancer-IgG的表达量呈正相关。本研究在GEO、TCGA和GTEx数据库中发现IGHG1在NSCLC中的表达量显著高于正常肺组织，并且高表达IGHG1的NSCLC患者预后较差。目前商业化的抗IgG1抗体是用人外周血提取的IgG做为免疫原获得的，不能区分B细胞来源的及肿瘤来源的IgG，我们及前期的报道已表明，用商品化抗人IgG进行免疫组化染色时，肿瘤细胞及间质细胞都有阳性反应。RP215抗体是用肿瘤细胞裂解液作为免疫原获得的，可特异性识别肿瘤来源免疫球蛋白重链恒定区的一个特殊唾液酸化表位，而不识别B细胞来源的IgG^[9]^。所以RP215对肿瘤来源的IgG更具有特异性。故本研究进一步利用RP215免疫组织化学染色的方法发现cancer-IgG在NSCLC中的表达水平显著高于正常肺组织，低表达cancer-IgG患者总生存期显著长于高表达的患者，分析患者临床特征发现cancer-IgG与NSCLC患者的临床分期、T分期和转移相关。

GSEA结果显示，高表达IGHG1样本富集到了细胞黏附、细胞因子-细胞因子相互作用和趋化因子信号通路。细胞黏附分子（cell adhesion molecules, CAM）是细胞与细胞、细胞与细胞外基质之间黏附作用和信息传递的膜蛋白受体，存在于正常细胞中维持组织正常形态和功能。CAM表达异常，可导致肿瘤细胞间黏附能力降低，侵袭转移能力增强^[24]^。也有研究^[25]^表明，CAM通过调节血管内皮细胞的功能，来促进肿瘤血管的形成，导致肿瘤的复发和转移。细胞因子-细胞因子相互作用和趋化因子信号通路在调节细胞生长分化、细胞间相互作用、免疫调节和肿瘤转移方面发挥重要的作用^[26, 27]^。高表达IGHG1样本富集到的基因集都参与了肿瘤的侵袭和转移，提示IGHG1可能通过增加肿瘤细胞的侵袭转移能力，来影响NSCLC患者的疾病进程。但是，该分析结果基于mRNA表达水平，并不能完全代表信号通路蛋白水平的改变，应进一步结合基础实验，验证该假设。

综上所述，cancer-IgG可能通过细胞黏附、细胞因子-细胞因子相互作用和趋化因子信号通路影响肺癌细胞的侵袭和转移，并且可以作为评价NSCLC患者预后的指标。
